# Advanced Diagnostic Approaches for Necrotrophic Fungal Pathogens of Temperate Legumes With a Focus on *Botrytis* spp.

**DOI:** 10.3389/fmicb.2019.01889

**Published:** 2019-08-14

**Authors:** Marzia Bilkiss, Muhammad J. A. Shiddiky, Rebecca Ford

**Affiliations:** ^1^School of Environment and Science, Environmental Futures Research Institute, Griffith University, Nathan, QLD, Australia; ^2^Queensland Micro- and Nanotechnology Centre (QMNC), Nathan, QLD, Australia

**Keywords:** Botrytis gray mold, biosensor, diagnosis, legumes, nanotechnology, nano-biosensor

## Abstract

Plant pathogens reduce global crop productivity by up to 40% per annum, causing enormous economic loss and potential environmental effects from chemical management practices. Thus, early diagnosis and quantitation of the causal pathogen species for accurate and timely disease control is crucial. Botrytis Gray Mold (BGM), caused by *Botrytis cinerea* and *B. fabae*, can seriously impact production of temperate grain legumes separately or within a complex. Accordingly, several immunogenic and molecular probe-type protocols have been developed for their diagnosis, but these have varying levels of species-specificity, sensitivity and consequent usefulness within the paddock. To substantially improve speed, accuracy and sensitivity, advanced nanoparticle-based biosensor approaches have been developed. These novel methods have made enormous impact toward disease diagnosis in the medical sciences and offer potential for transformational change within the field of plant pathology and disease management, with early and accurate diagnosis at the point-of-care in the field. Here we review several recently developed diagnostic tools that build on traditional approaches and are available for pathogen diagnosis, specifically for *Botrytis* spp. diagnostic applications. We then identify the specific gaps in knowledge and current limitations to these existing tools.

## Introduction

Legumes are the member of third largest plant family, Fabaceae, which comprises over 20,000 species are grown in a broad spectrum of climatic conditions and soil categories (Cernay et al., 2016). Legume crops help to build soil fertility by fixing nitrogen, act as a disease break for cereal and oilseed crops are staple foods, providing a significant source of protein, dietary fiber, carbohydrates and dietary minerals, as well as essential amino acids. Several represent high value cash export food crops, together representing 27% of the total global crop production (Smýkal et al., 2015).

Grain legumes are susceptible to attack by numerous pathogens, including bacteria, fungi, viruses to nematodes and parasitic plants, resulting in large global economic disaster. In particular, necrotrophic fungal pathogens such as *Ascochyta* spp., *Botrytis* spp., *Colletotrichum* spp. and others cause widespread disease and severe crop loss. Botrytis gray mold (BGM) of temperate grain legumes is caused by the fungal species *Botrytis cinerea* and *Botrytis fabae* separately or in a complex. The disease occurs particularly in dense, early-sown crops, when there are prolonged wet periods. The fungus can affect all above ground plant parts, resulting in tissue necrosis, flower and pod abortion, and seed shriveling and marking^1^. The loss in faba bean (*Vicia faba* L.) and lentil (*Lens culinaris* Medik.) from attack from *Botrytis* spp. in Australia alone during a conducive year is around AUD $1.4 million and AUD $1.0 million, respectively (Murray and Brennan, 2012).

Large economic and environmental savings could be made through early, fast and accurate diagnosis of the pathogen when the inoculum is in sufficient levels to cause an epidemic. This would lead to strategic timing and potential reduction in amounts and/or numbers of fungicide applications. To date, several immunogenic and molecular probe-type diagnostic methods have been developed for *B. cinerea* (Gachon and Saindrenan, 2004; Suarez et al., 2005; Chilvers and Lindsey, 2006; Rigotti et al., 2006; Diguta et al., 2010; Fernández-Baldo et al., 2011; Duan et al., 2014; Fan et al., 2015). However, their ability to discriminate between *Botrytis* ssp. and hence their usefulness in the paddock is unclear. Meanwhile, these existing methods, together with those based on whole-genome-sequencing and polymerase chain reaction (PCR) amplification are time-consuming and offer varying levels of sensitivity (Fan et al., 2015).

## Necrotrophic Fungal Pathogens of Grain Legumes

Necrotrophic fungi kill the plant tissue during invasion and subsequently live saprotrophically on the dead cells. Important temperate grain legumes such as chickpea (*Cicer arietinum* L.), lentil, field pea (*Pisum sativum*) and faba bean are impacted by major necrotrophic fungi belonging to the *Ascochyta* spp., *Botrytis* spp., *Colletotrichum* spp. and *Phoma* spp. Together, these represent a $1.9 million per year loss in lentil, a $2.9 million per year loss in faba bean, a $5.2 million per year loss in chickpea and a $20.0 million per year loss in field peas (Murray and Brennan, 2012). Losses occur through direct seed loss and reduced marketability as well as disease management including chemical and cultural methods and through to the cost of breeding for resistant cultivars. Disease management costs would be reduced through rapid, sensitive and accurate pathogen identification. Additionally, several of the above-mentioned pathogens infect across-species, have similar disease symptoms on the plants and may be difficult to discriminate under a microscope due to very similar conidia spore morphology Therefore, a faster and highly accurate, species-specific, diagnosis would effectively enable a faster and more accurate response for management.

### Diagnostic Methods Currently Used for Necrotrophic Fungi of Temperate Grain Legumes

Traditional diagnostic methods are mainly based on visual inspection of structural characters like shape and size of conidia, spores and cultural characters like growth rate and colony color (Taylor and Ford, 2007). However, these methods are relatively slow, often taking over a week to obtain as well as requiring a skilled pathologist to reliably identify the organism. Rapid diagnosis is required for the appropriate disease control measures to prevent an epidemic within an entire crop.

More recently, diagnostic assays for plant pathogens have been developed using nucleic acid sequences. Molecular approaches, mainly PCR-based, have been used extensively for fungal pathogens detection (Martin et al., 2000; Schaad and Frederick, 2002). Phan et al. (2002) designed a selective and sensitive diagnostic marker to differentiate *Ascochyta rabiei* of chickpea from other host specific *Ascochyta* spp. using ribosomal nucleotide sequence data with a detection limit of 0.1 pg of pathogen DNA.

Advanced molecular detection techniques such as the real time polymorphic chain reaction, loop mediated isothermal amplification (LAMP) method have also been developed (Notomi et al., 2000). Bayraktar et al. (2016) developed a real time PCR method to detect down to 1 pg of *A. rabiei* gDNA, a limit of just 0.1 pg of *A. rabiei* gDNA was detected with a LAMP assay, with the advantage of the visualization of color change with the naked eye (Chen et al., 2016).

## Case Study: Detection of *Botrytis* spp. of Temperate Grain Legumes

### Incidence and Global Impact of Botrytis Gray Mold Diseases on Grain Legumes

The major staple grain legume, chickpea, is impacted by the foliar disease BGM, also known as chocolate spot (Pande et al., 2009). The disease on chickpea is attributed solely to *B. cinerea* and the first occurrence was noted in India by Shaw and Ajrekar (1915) subsequently by Butler and Bisby (1931). The disease remains a major issue in Australia, Argentina, Bangladesh, India, Nepal, and Pakistan (Davidson et al., 2007) where complete crop losses were reported under conducive environmental conditions (Pande et al., 2009). BGM on chickpea were also been reported in Colombia, Chile, Canada, Mexico, Hungary, Spain, Myanmar, Turkey, and Vietnam (Pande et al., 2006).

Botrytis Gray Mold is also a major constraint to lentil production in Australia, Nepal, Bangladesh, Canada, Pakistan, Colombia, New Zealand, India, Morocco, Syria, and the United States (Davidson et al., 2007). On lentil, the disease is caused by co-existing and unknown mixed ratios of *B. cinerea* and *B. fabae* (Lindbeck et al., 2008, 2009). In Australia, severe epidemics with losses exceeding 50% were first reported in 1999 (Davidson et al., 2007). Similarly, BGM on faba bean is caused by both *B. cinerea* and *B. fabae* with yield losses in excess of 90% (Beyene et al., 2016). BGM also impacts production of several other grain legumes including field pea (Kraft and Pfleger(eds), 2001) and mung bean (Li et al., 2016).

### Management and Control of Botrytis Gray Mold (BGM) on Grain Legumes

Botrytis Gray Mold is difficult to manage since the causal pathogens are soil, seed, and air borne (Davidson et al., 2007). Extensive screening of chickpea and lentil germplasm for reaction to the fungi has failed to identify high levels of resistance (Pande et al., 2006; Davidson et al., 2007). Similarly, only moderate levels of quantitative resistance have been reported in faba bean (Bouhassan et al., 2004; Tivoli et al., 2006). Therefore, fungicides remain a major component of BGM disease management strategy. However, these are often used only after symptoms are visible, at which point a substantial epidemic may already be well underway, requiring multiple subsequent foliar sprays at regular intervals.

Frequent use of fungicides can lead to the development of isolate population with loss of sensitivity to chemicals with multiple modes of action and the spread of these isolates is of huge concern to the horticultural and cropping industries (Rupp et al., 2017). Also, prolonged, repeated and over-spraying potentially through lack or misdiagnosis of the causal pathogen, can cause environmental contamination with substantial toxicological concern (Komárek et al., 2010). Informed timing of application, optimally prior to symptomology and in combination with climatic predictors greatly enhances fungicide efficacy (Eikemo et al., 2013). Indeed, early detection of the causal agents of BGM remains the most important step in effective and targeted management of the disease in the field and in greenhouse systems (Mertely et al., 2002).

### Diagnostics for *Botrytis* spp.

As necrotrophic fungi *Botrytis* ssp. can survive from one season to another and are readily dispersed by wind or water. Infections are recognizable by their gray conidial structures within infected crop canopies but only once an advanced epidemic is underway. Several methods have been developed to identify these fungal species as follows:

#### Traditional Methods

*Botrytis* species have traditionally been identified through non-precise, time-consuming and non-sensitive visual symptomology, plate culturing and histopathological methods (Dewey and Yohalem, 2007). The later stages of infection by Botrytis spp. are easily recognized followed by the gray and/or brown conidial structures on the surface of infected host (Khazaeli et al., 2010). However, the initial or dormant and endophytic stages of infections are difficult to detect in non-symptomatic material (Molly and Grant-Downton, 2015). Also, the morphologies of conidiospores of *Botrytis* ssp. are often indistinct (Harrison, 1988; Beever and Weeds, 2007) and the use of a microscope is somewhat impractical for field diagnostics (Ray et al., 2017).

#### Immunoassay-Based Diagnostics

Several immunoassay methods have been developed to diagnose and quantify to a lower levels of plant pathogens. By far the most commonly used method for *Botrytis* ssp. species has been enzyme-linked immunosorbent assays (Dewey and Yohalem, 2007). By raising the antisera in rabbits, Ricker et al. (1991) applied plate-trapped antigen-immunosorbent assays to determine levels of *Botrytis* antigens in infected grapes juice. The same protocol, with the genus-specific monoclonal antibody (MAB) BC-12.CA4, was used to detect and quantify *B. cinerea* in pear stems (Meyer et al., 2000), strawberries (Mehli et al., 2005), grape berries (Obanor et al., 2004), raspberries (Dewey et al., 2000) and latent *B. aclada* infections in onion leaves (Yohalem et al., 2004). Production of MAB for *Botrytis* spp. is now somewhat routine^2^. The laboratory-based plate-trapped antigen ELISA (Meyer et al., 2000)was time-consuming, involving an overnight antigen coating step with extracts from infected plant tissues followed by sequential incubations with a monoclonal antibody, a secondary enzyme conjugated anti-mouse antibody and a substrate with washings between each step (Molly and Grant-Downton, 2015). By increasing the concentration of the secondary reporter antibody, the total time involved for diagnosis was subsequently reduced to 20 min (Dewey et al., 2000). Meanwhile, a microtiter immune-spore trapping device (MTIST device), that uses a suction system to directly trap air particulates by impaction in microtiter wells., was used together with the BC12.CA4 antibody for rapid detection and quantification of *B. cinerea* (Kennedy et al., 2000), Fernández-Baldo et al. (2011) then used BC-12.CA4 within a competitive ELISA quantification of *B. cinerea* in apple, table grape and pear tissues. In this assay, *Botrytis*-ssp. antigens present in extracts from infected tissues compete with “purified” *B. cinerea* antigens immobilized on the surface of microtiter plates using a cross linking agent. Although the assay takes about 40 min with a detection limit of 0.97 μg/ml, including in non-symptomatic infections, the BC12.CA4 antibody was subsequently shown not to discriminate among *Botrytis* species (Binder, 2014).

#### Molecular-Based Diagnostics

As an alternative to traditional and immunogenic approaches, unique diagnostics for individual *Botrytis* spp. may potentially be developed using molecular capture probes designed to individualized targets (DNA sequence in the first instance), that are stable and robust in terms of evolutionary potential. Accordingly, several nucleic acid-based probe targets have been described for the detection of *B. cinerea* including to the ribosomal intergenic spacer (IGS) region (Suarez et al., 2005), β-tubulin (Brouwer et al., 2003; Mehli et al., 2005; Spotts et al., 2008), cutinase A (Gachon and Saindrenan, 2004) and RNA helicase (Celik et al., 2009) genes, as well as *ad hoc* sequence characterised amplified region (SCAR) sequences (Rigotti et al., 2002; Suarez et al., 2005; Cadle-Davidson, 2008). However, the use of some ribosomal sequences for diagnostic purposes have been criticized due to non-stable inheritance (Suarez et al., 2005) and assays targeting specific genes like the cutinase A and β-tubulin from *B. cinerea* have been characterized to cross-react with close relatives such as *B. fabae* (Suarez et al., 2005; Spotts et al., 2008).

Depending on sequence deviation in the nuclear ribosomal IGS region Chilvers et al. (2007) developed a real-time PCR assay with primer set, LR12R/CNS1, for the quantification of *Botrytis aclada*, *Botrytis allii*, and *Botrytis byssoidea* in onion seed. Subsequently a TaqMan real-time PCR assay was developed by Carisse et al. (2009) with the primer set, B_squa_up221/B_squa_lo361. This was used to quantify the conidia of *Botrytis squamosa*, causal agent of botrytis leaf blight of onion. Similarly for diagnosis of *B. cinerea* on grapes, Diguta et al. (2010) developed a real-time PCR assay was based on IGS sequence with a detection limit of 6.3 pg DNA (corresponding to ∼540 genomes). Meanwhile, *Botrytis fabiopsis*, a newly characterized species causing BGM on broad bean in central China, was identified (Zhang et al., 2010). Fan et al. (2015) used three primer sets, Bc-f/Bc-r, Bfa-f/Bfa-r, and Bfab-f/Bfab-r, from the necrosis and ethylene-inducing protein 1 (NEP1) gene, to discriminate *B. cinerea*, from *B. fabae*, and *B. fabiopsis* in broad bean. These were species-specific in both single and multiplex PCR assays with the detection limits of 40, 40, and 400 pg (∼869, ∼869, and ∼8690 genomes) of purified gDNA, respectively. Furthermore, the existence of broad bean gDNA in the PCR reaction at 1:1000 (*Botrytis* gDNA/broad bean gDNA (wt/wt) had insignificant effect on diagnosis of the presence of *Botrytis* spp. The multiplex PCR assay could detect all the three *Botrytis* spp. in both artificially and naturally infected broad bean leaves (Fan et al., 2015).

To improve the portability of molecular diagnostic assays, a DNA amplification technique known as LAMP was developed, which amplifies nucleic acid with high selectivity, sensitivity and quickly under isothermal conditions (Notomi et al., 2000). LAMP products can be visualized with the naked eye by adding DNA-intercalating dyes, metal-ion indicators (Goto et al., 2009), CuSO_4_ (Zoheir and Allam, 2011), calcein (Tomita et al., 2008) or by measuring the increase in turbidity derived from magnesium pyrophosphate formation to infer increases in amplified DNA concentration (Mori et al., 2001). LAMP products may be assessed in a real-time detection format on a portable, low power platform, such as Genie I (OptiGene, United Kingdom) (Tomlinson et al., 2010). LAMP probes have been designed for *B. cinerea* targeting the ribosomal IGS (Tomlinson et al., 2010) with a detection limit of 6.5 pg (∼140 genomes) using hydroxynaphthol blue dye (HNB). LAMP assays for *B. cinerea* have also been developed based on the Bcos5 gene with a detection limit of 10^–3^ ng/mL (∼2.17 genomes; Duan et al., 2014) and was patented (CN103276057A;CN104293957A). Although, diagnostic methods using molecular probes alone have proven to be rapid and have high specificity and sensitivity, there remain limitations in detecting pathogens at low titers in materials at early infection stages (Beever and Weeds, 2007; Herbert et al., 2008; Khater et al., 2017). Also, false positive results are possible through cross-amplification of PCR-generated fragments of non-targets not considered during assay development. Further, false negatives may result from mis-amplification of target DNA (Khater et al., 2017). Consequently, further improvements to the robustness of molecular diagnostics systems are needed, which may include the use of biosensor devices.

## Biosensor-Advanced DIAGNOSTIC APPROACHES FOR RAPID, SPECIFIC AND SENSITIVE PATHOGEN DIAGNOSTICS

A biosensor is an analytical device used to detect target molecules by producing a readout signal proportional to the specific interaction of an analyte with a recognition element (Mascini and Tombelli, 2008). In recent years, biosensors have been broadly used as diagnostic tools in environmental, food and clinical analysis, as well as in plant pathogen diagnosis and analysis (Mehrotra, 2016; Khater et al., 2017). Different types of sensor formats have been utilized for pathogen diagnosis and analysis of various biological elements at the level of tissue, microorganism, cell receptor, organelle, enzyme, antibody and nucleic acid. This has created new opportunities for highly sensitive protein detection in the cancer industry (Chikkaveeraiah et al., 2012; Rama and Costa-García, 2016; Marques et al., 2018), for detecting food borne pathogens (Wang et al., 2012; Xu et al., 2016), and biological and chemical contaminants in the agricultural sector (Linting et al., 2012; Tran et al., 2012; Regiart et al., 2017).

### Biosensor Technologies Used in the Agricultural Sector

Electrochemical immunoassays with nanostructured surfaces, nanoparticle labels and magnetic beads have created new opportunities for thoroughly sensitive detection of biological and chemical contaminants in the agricultural sector (Linting et al., 2012; Tran et al., 2012; Regiart et al., 2017). In this device, an electrochemical immunosensor produces an electroactive signal that is detected by transducers through the use of an antibody that is incorporated into a bio-recognition layer to generate a measurable signal. Label-free electrochemical immunosensors with acceptable stability and reproducibility have been applied for pesticide analysis in crop samples (Tran et al., 2012; Liu et al., 2014). For the detection of atrazine, a widely used pesticide in agriculture, Liu et al. (2014) developed a simple, specific, sensitive electrochemical immunosensor that is characterized by cyclic voltammetry (CV) and electrochemical impedance spectroscopy (EIS) using ferricyanide as an electrochemical redox indicator. This biosensor uses immobilized gold nanoparticles (GNPs) on a gold electrode surface and under favorable condition the limit of detection for atrazine is just 0.016 ng/mL (Liu et al., 2016).

As an electroactive label, horseradish peroxidase (HRP) exhibits good stability and solubility to catalyze substrates to produce quantitative electrochemical signals (Zhao et al., 2011). By utilizing the favorable conductivity of the large surface area of gold nanoparticles (AuNPs) and high-quality catalytic ability of HRP, a dual amplified electrochemical immunosensor has been developed for accurate and sensitive diagnosis of *Pantoea stewartii* subsp. *Stewartia* of maize, with a detection limit of 7.8 × 10^3^ cfu/mL. This was 20-fold more sensitive than conventional ELISA (Zhao et al., 2011).

Meanwhile, glass-poly(dimethylsiloxane) (PDMS) microfluidic sensors comprise a glass substrate that provides a thermally stable and amenable platform for sensing of a target molecule by deposition of the electrodes by sputtering (Hatamie et al., 2015). For these biosensors, zinc oxide nano rods are fabricated on non-commercial electrodes and the glass substrate is coated with a gold layer. The sensing is then determined through voltammetric changes during antibody capture. This type of biosensor has the advantages of: low cost, durability, chemical inertness, optical transparency and automation (Casanova-Moreno et al., 2017). By utilizing this technique, the bacterial pathogen *Xanthomonas arboricola* was detected very quickly and at 1.5 × 10^2^ cfu/mL in walnuts, based on the covalent immobilization of a MAB (Regiart et al., 2017).

Optical biosensors offer the advantage of label-free real time detection of chemical and biological substances by exploiting the interaction of an optical field with a bio-recognition element (Damborský et al., 2016). The central point of this method is the ability to detect the target microorganism via color change, without any analytical instrument. This has immense portability and the generation of an instant result. However, the major drawback is low sensitivity, for example a detection limit of just 5 × 10^4^ cfu/mL for *Escherichia coli* (Pöhlmann et al., 2014) compared to the limit of detection by electrochemical assay of 32 cfu/mL (Li et al., 2015).

Fluorescence-based immunosensors are used to measure the concentration and intensity of target analyte fluorescence to which fluorescent molecules bind directly or indirectly (Holford et al., 2012). For this, an external short-wavelength light source is required to initiate electronic transitions in a molecule for producing longer wavelengths of light. The fluorescent bioreceptor and optical transducer combination was used in the fluorescent-based optical biosensor to diagnose *Phytophthora palmivora*, *Ustilago maydis*, *Colletotrichum lindemuthianum*, *Aspergillus nidulans* like plant pathogenic fungi (Ray et al., 2017). Charlermroj et al. (2013) used this technique to develop a microsphere immunoassay to detect important plant pathogens like, watermelon silver mottle virus (WSMoV), fruit blotch bacterium (*Acidovorax avenae* subsp. *Citrulli*), melon yellow spot virus (MYSV) and chilli vein-banding mottle virus (CVbMV). Antibody correlated to fluorescence-coded magnetic microsphere for each plant pathogen used to capture corresponding pathogen. The presence of the pathogens were identified by secondary R-phycoerythrin (RPE)-labeled antibodies with the limit of detection of 6 × 10^5^ cfu/ml, 1.0 ng/ml, 20.5 ng/ml and 35.3 ng/ml, respectively (Charlermroj et al., 2013). Though this assay showed relatively sensitivity, it requires a multi-channel fluorescence reader (Khater et al., 2017). Gold nanoparticle/multi-walled carbon nanotube (Nano-Au/C-MWCNT) based label free immunosensor was used to detect capsicum chlorosis virus (CaCV) in bell pepper which showed 800–1000 times more sensitivity than ELISA (Sharma et al., 2017).

Biosensors have been developed to detect pathogens through surface enhanced Raman spectroscopy (SERS) and surface plasmon resonance (SPR) (Tokel et al., 2014; Yoo and Lee, 2015). The SPR sensor technology uses electromagnetic waves that can be stimulated by light at gold sensor surface (Skottrup et al., 2008). The incoming light, associate with the gold interface, at angles greater than the critical angle and the reflected light exhibits a characteristic devaluation due to resonant energy transfer from the incoming photons to the surface plasmons (Skottrup et al., 2008). Skottrup et al. (2008) compared SPR-based biosensors for the detection of plant pathogens and noted a diversity in sensitivity level including those developed for *Fusarium culmorum*, *Puccinia striiformis* and *Phytophthora infestans* at 0.06 pg/ml (∼2 spores/ml), 3.1 × 10^5^ spores/ml and 2.2 × 10^6^ spores/ml, respectively (Skottrup et al., 2007a, b; Torrance et al., 2006; Zezza et al., 2006, respectively). Meanwhile, Mendes et al. (2009) reported a SPR-based immunosensor for detecting soybean rust on leaves whereby an antibody to *Phakopsora pachyrhizi* was covalently immobilized on a gold substrate via a self-assembled monolayer (SAM) of thiols using cystamine-coupling chemistry. This biosensor had a detection limit of 8 μg/ml (9.26 × 10^6^ spores/ml). The ability of real-time and label-free detection of targets is the major advantage of using SPR in pathogen detection, however, low sensitivity and specificity due to the high non-specific adsorption of non-targets onto the sensor surface remains a major hurdle.

Accordingly, SERS has been used in various biosensor-based pathogen detection techniques because of its single molecule-level sensitivity, molecular selectivity and quenching insensitivity (Yoo and Lee, 2016). This method provided a 10–100 times narrower spectral widths than previous methods and minimized overlapping between different labels (Bantz et al., 2011). Gold nanoparticle probes with versatile Raman dyes have also become popular for development of SERS-based immunoassays (Xu et al., 2004).

Although the power of nano-biosensors for the timely, accurate and at-point-of-contact diagnostics is clear, there remain major challenges to their use that are related to the isolation and capture of specific targets from biological and environmental backgrounds and in the way in which the target capture is relayed as a signal of detection.

## Challenges Involved in Developing an *In-Field* Nano-Biosensor for *Botrytis* spp.

### Biological Challenges

#### Sampling and *In-Field* Processing

Though biosensors showed higher sensitivities compared to traditional, immunogenic or molecular assays, majority have been tested with synthetic samples and in *in vitro*. Many are yet to be validated in field, which incorporates optimization of target extraction from the host tissue (Ray et al., 2017). A quick sample processing of samples is also a requirement. The BGM target will be fungal spores and/or mycelia, dependant on the stage of infection or epidemic. Airborne spores are the major source of inoculum generally blown in through prevailing air currents from neighboring crops. However, the pathogens also survive between seasons as hardened bodies of mycelial sclerotia in the soil and on infected plant trash (previously harvested stems) (Lindbeck et al., 2009). Therefore, careful consideration must be given to capturing the target organism, potentially via monitoring and surveillance using aerial trapping or screening of plant surfaces. Consideration should also be given to determining how many spores/genomes of a pathogen are required to lead to a disease epidemic under optimal climatic and host conditions to ensure that a quantitative diagnostic is realistically translatable to informing disease management decision (González-Domínguez et al., 2015).

Consideration should be given to the potential for biological interference from the sample used for diagnosis. In the case of BGM, the pathogen is both external and internal to the host plant tissue, existing as mycelia on the foliage and as conidiophores within foliar lesions at later stages of the infection cycle. If the diagnostic is applied to an infected plant sample which is then used as the complete sample (containing both fungus and plant), the assay may be impacted by the noise of the plant itself which contains a variety of nucleic acids and polymers. These plant cell components have the potential to cause subsequent loss of sensitivity with diagnostic processes from inaccurate or reduced molecular probe binding as well as causing electromagnetic interference (McCann et al., 1997). Also, if using a molecular probe, potential co-extraction of high content of tannins, polysaccharides, and polyphenols may lead to oxidation and degradation of the target fungal gDNA sequences, again leading to failure or loss of detection sensitivity (Arruda et al., 2017).

### Technical Challenges

Several pre-analytical steps involved in DNA, RNA, or whole pathogen isolation and detection may significantly affect the sensitivity and accuracy of a nano-biosensor as follows:

#### Background Interference

In many assay designs, target nucleic acids are initially captured and isolated by a pre-isolation step where they are hybridized to a complementary capture probe. However, the capture of a specific nucleic acid (DNA or RNA) target is greatly reduced in accuracy when present in a background that is populated with highly homologous non-target sequences, i.e., plant sequences from the same family as the target fungal sequence. Accordingly, probes must be exact and able to robustly differentiate even single nucleotide polymorphisms (SNPs). The biological challenge from highly-heterologous samples may also result in the technical challenge of non-specific absorption of molecules to the sensor surface inducing false responses (Islam et al., 2017). Therefore, a suitable blocking/antifouling agent such as mercapto-ethanol, poly (ethylene glycol), mercaptohexanol or bovine serum albumin is often used (Gooding, 2002; Islam et al., 2017). Electro biochemical sensors are also impacted by the potential non-specific adsorption of non-target nucleic acids onto the electrode surface (Lucarelli et al., 2004), which can lower hybridization efficiency to less than 10% (Levicky et al., 1998). Again, this is greatly improved by exposing the DNA modified surface to a blocking agent (Gooding, 2002; Islam et al., 2017).

#### Target Capture Efficiency

The dynamics of the specific nucleic acid hybridization plays a crucial role in the specificity and sensitivity of a hybridization-based DNA nano-biosensor (Liu et al., 2012). The capture probe, which is complementary to the single-stranded target sequence, detects the target gDNA by hybridization onto the recognition interface (Gooding, 2002). For this to be accurate, the probe sequence must be designed unique to the target. During the hybridization, if the capture probe and the target sequences are not permitted to coil around each other, if so they will not hybridize efficiently (Gooding, 2002). Some other factors such as end point immobilization of probe strand (Levicky et al., 1998), density and surface chemistry of probe strand (Gooding, 2002) also play key roles in determining the hybridization efficiency.

#### Limits of Probe Specificity and Sensitivity

Different construction of capture probes affects the hybridization efficiency of biosensor which is an important factor in the specificity and sensitivity of the biosensor (Liu et al., 2012). Ranging 18–25 nucleotides, usually allows higher level of specificity in hybridization reaction where excessive length of capture probe often results unfavorable yields (Lucarelli et al., 2008). Due to the spatial hindrance effect, a probe cannot capture a target analyte if the density is too low or too high (Ding et al., 2013). Therefore, surface coverage of the probe DNA is a crucial issue. Too few binding sites (<10^–12^ cm^–2^) will result in a reduced detection sensitivity.

## Biosensors Developed for *Botrytis* spp. Detection

### Electrochemical Biosensors

**FIGURE 1:**
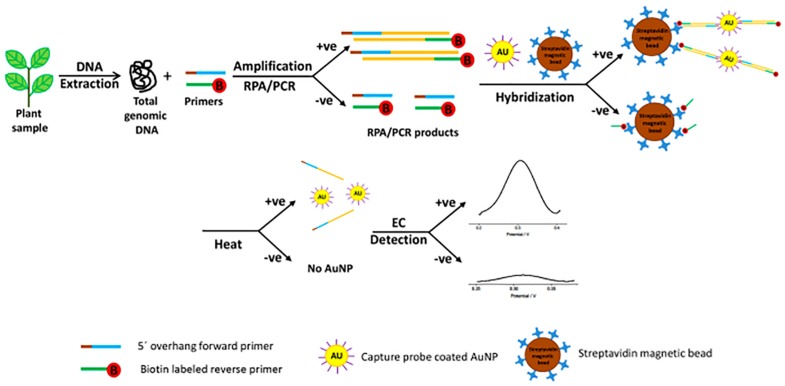
Schematic representation of the electrochemical bioassay for plant pathogen diagnosis involving incubation of the amplified target, AuNPs-DNA probes and streptavidin beads (reproduced with permission from Lau et al., 2017).

For *Botrytis* spp. detection in onion, Binder (2014) developed an electrochemical immunosensor using screen printed gold electrodes (SPGE) primed with horseradish peroxidase (HRP) that were able to detect 34 ng/ml (∼7 × 10^5^ spores/ml). Most recently, a fast (60 min) and sensitive (214 pM or ∼3 × 10^8^ spores/μl) diagnostic protocol was developed for the detection of *Pseudomonas syringae* from infected plant samples via rapid isothermal amplification of target sequences by RPA followed by gold nanoparticle-based electrochemical stimulus with differential pulse voltammetry (DPV) (Lau et al., 2017; Figure 1). Briefly, the isothermally amplified targets were first incubated with the AuNPs/DNA probe. The mixture was then incubated with streptavidin-coated magnetic beads. After performing a magnetic collection of the beads, the mixture was resuspended in PBS and denatured at 95°C and following a second magnetic separation, the supernatant was transferred to an electrode surface for DPV signal capture.

### Optical Biosensors

**FIGURE 2:**
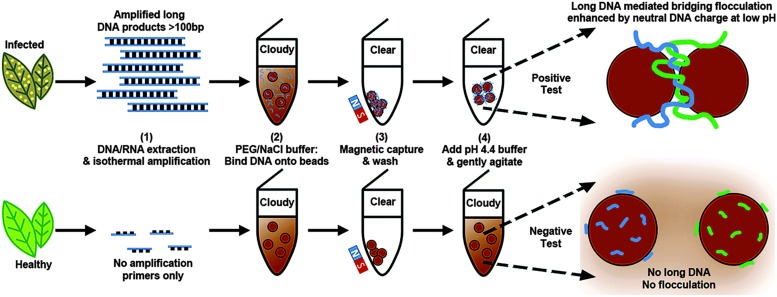
Schematic representation of the DNA-based bridging flocculation assay (reproduced with permission from Wee et al., 2015).

A novel assay was developed to detect isothermally amplified DNA of important plant pathogens, including *Botrytis* spp. (Wee et al., 2015). The DNA-mediated bridging flocculation assay develops to detect the presence of the target pathogen gDNA, which is amplified to produce high molecular weight DNA amplicons (Figure 2; Wee et al., 2015). This method provided a yes/no pathogen presence result for immediate informed disease risk management and was used to detect *B. cinerea* at very early infection stages, when symptoms were just visible to the naked eye with the detection limit of less than 0.3 pg/μl (∼ 6 spores/μl) (Wee et al., 2015).

**FIGURE 3:**
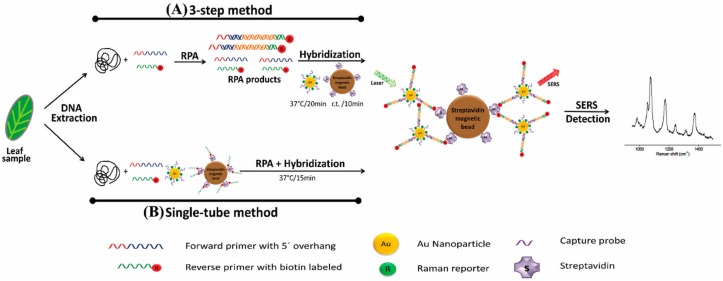
Schematic representation of RPA/SERS multiplex assay (reproduced with permission from Lau et al., 2016). **(A)** Genomic DNA was extracted using modified Solid Phase Reversible Immobilization (SPRI) method, followed by recombinase polymerase amplification (RPA) using specific biotinylated capture probe. After amplification, biotin/RPA/SERS products were captured by streptavidin magnetic beads. **(B)** For faster and simpler assessment, SERS nanotags were amplified and hybridized in a single tube.

Recently, *B. cinerea*, *P. syringae*, and *Fusarium oxysporum* were detected in *Arabidopsis thaliana* and tomato plants via a multiplex point-of-care diagnostic platform developed using a combination of SERS and RPA (Figure 3; Lau et al., 2016). The method was faster and 100 times more sensitive than PCR alone and could detect just two *B. cinerea* genomes.

Although showing rapid and accurate result, a large limitation of SERS biosensors is the requirement for bulky optical instruments, such as lasers, optical microscopes, detectors and monochromators (Yoo and Lee, 2015). These do not translate well into the field environment. Therefore, scope remains for development of a rapid, accurate, inexpensive and sensitive nano-biosensor for plant pathogens.

## Summary and Conclusion

There are a range of technologies used in advance diagnostic assays for plant pathogens. Each of these has advantages and biological and technical limitations that relate to specificity, sensitivity, speed to result, quantification, portability, and cost. Recent advances in electromagnetic nano-biosensors coupled with species-specific molecular probes has the potential to overcome many of these limitations. Ultimately, a practicable diagnostic method would instantly inform growers on the presence and quantity of the target pathogen and be able to accurately detect at the threshold of spore density to create an epidemic. Unfortunately, the currently even the most advanced Botrytis species diagnostics do not meet these criteria, being either non-specific or lacking in sensitivity, and/or unable to provide a portable and instant result (Table 1). Therefore, the challenge remains to provide such a tool to the cropping industries at a cost low enough to be commercially viable for wide uptake to guide informed disease management, specifically for necrotrophic fungal pathogens to aid in the use and timing of fungicide application.

**TABLE 1:** Summary of published diagnostic methods and their detection limits for crop disease-related *Botrytis* species.

***Botrytis* species**	**Detection Technique**	**Description**	**Detection Limit**	**References**
*B. aclada, B. allii, B. byssoidea, B. cinerea and B. squamosa*	PCR	PCR detection and RFLP differentiation of *Botrytis* spp.	1 to 10 pg DNA	Nielsen and Protection, 2002
*B. cinerea*	PCR	Conventional PCR detection from a unique PCR fragment obtained by RAPD analysis in strawberry	2 pg DNA	Rigotti et al., 2002
*B. fabae, B. fabiopsis and B. cinerea*	PCR	PCR detection and differentiation of three species in broad bean for chocolate spot	40 pg, 40 pg and 400 pg of DNA, respectively	Fan et al., 2015
*B. aclada, B. allii and B. byssoidea*	Real-time PCR	Real-time quantitative PCR assay for estimation of the pathogen load in onion seeds	0.01 pg of DNA	Chilvers et al., 2007
*B. aclada*	Real-time PCR	Real-time quantitative PCR assay for estimation of the pathogen load in onion bulb tissue	0.01 pg of DNA	Coolong et al., 2008
*B. cinerea*	Real-time PCR	Real-time PCR assays based on TaqMan chemistry	0.02 pg to 20 pg DNA	Suarez et al., 2005
*B. cinerea*	Real-time PCR	Identification and quantification of *B. cinerea* in grapes	6.3 pg DNA	Diguta et al., 2010
*B. cinerea*	LAMP	LAMP assay based on intergenic spacer of the *B. cinerea* nuclear ribosomal DNA	6.5 pg DNA	Tomlinson et al., 2010
*B. cinerea*	LAMP	LAMP assay based on the Bcos5 sequence	1 pg DNA	Duan et al., 2014
*B. cinerea*	Immunosensor	Quantitative competitive immunosensor for the diagnosis of *B. cinerea* in fruits (tested with pears, apples, grapes).	8 × 10^3^ pg DNA	Fernández-Baldo et al., 2010
*B. cinerea, B. allii*	Immunosensor	Electrochemical immunosensor conjugated with HRP for *Botrytis* spp. detection using screen-printed gold electrodes (SPGE)	3.4 × 10^4^ pg DNA	Binder, 2014
*B. aclada*	ELISA	Indirect competitive ELISA (polyclonal Ab) for detection of *B. aclada* in onion bulbs.	Not specified	Linfield et al., 1995
*B. cinerea*	ELISA	Quantitative indirect ELISA (BC-12 Ab) for the detection of *B. cinerea* in fruits (tested with pears).	1 × 10^6^ to 1 × 10^7^ pg DNA	Meyer et al., 2000
*B. cinerea*	ELISA	Quantitative indirect competitive ELISA (BC-12 Ab) for the detection of *B. cinerea* in fruits (tested with apples, grapes).	9.7 × 10^5^ pg DNA	Meyer et al., 2000
*B. cinerea, B. allii*	ELISA	Commercially available B. cinerea (BC-12 Ab) and *B. allii* (polyclonal Ab) ELISA kits from ADGEN Phytodiagnostics (Auchincruive, United Kingdom).	1 × 10^6^ to 1 × 10^7^ pg DNA	Binder, 2014
*B. cinerea, Fusarium oxysporum* and *Pseudomonas syringae*	Electrochemical biosensor	A quick, specific and sensitive point of-care method for multiplex detection of plant pathogens by surface-enhanced Raman scattering (SERS) labeled nanotags and recombinase polymerase amplification (RPA)	0.1 pg DNA	Lau et al., 2017

## Author Contributions

All authors wrote and revised the manuscript, and approved it for publication.

## Conflict of Interest Statement

The authors declare that the research was conducted in the absence of any commercial or financial relationships that could be construed as a potential conflict of interest.

## Footnotes

1 http://www.pulseaus.com.au/about/australian-pulse-industry

2 http://dshb.biology.uiowa.edu/Botrytis-cinerea
